# Survivin expression and its clinical significance in pancreatic cancer

**DOI:** 10.1186/1471-2407-5-127

**Published:** 2005-10-04

**Authors:** Myung Ah Lee, Gyeong-sin Park, Hee-Jung Lee, Ji-Han Jung, Jin-Hyoung Kang, Young Seon Hong, Kyung Shik Lee, Dong-gu Kim, Seung-Nam Kim

**Affiliations:** 1 Division of Oncology, Department of Internal Medicine, Catholic University of Medical College, Seoul, Korea; 2 Department of Pathology, Catholic University of Medical College, Seoul, Korea; 3 Department of General Surgery, Catholic University of Medical College, Seoul, Korea

## Abstract

**Background:**

Survivin, an inhibitor of apoptosis is expressed in several human cancers. Its expression is known to be associated with poor clinical outcome, but not widely studied in pancreatic cancer. We performed this study to determine the survivin expression in pancreatic cancer and its clinical significance as a prognostic factor.

**Methods:**

We performed immunohistochemical staining for survivin, p53, and Bax in formalin-fixed, paraffin-embedded block from forty-nine pancreatic tissues. To determine the association with clinical course, we reviewed the patients' clinical record.

**Results:**

Of the 49 cases of pancreatic cancer, 46 cases (93.9%) were positive for survivin expression. There was no significant association between survivin expression and p53 or bax. For clinicopathological parameters, perineural invasion was more common in survivin positive and venous invasion was more common in survivin negative (p = 0.041 and 0.040, respectively). Responsiveness to chemotherapy appeared to be slightly better in patients with low survivin expression.

**Conclusion:**

Survivin expression may be associated with venous or perineural invasion, indicating metastatic route, and seems to have a potential as a predictive marker for chemotherapy. Further study of large scale is required to determine the clinical significance of survivin expression in pancreatic cancer.

## Background

Survivin has recently been identified as a novel inhibitor of apoptosis (IAP). It blocks common downstream elements of both the mitochondrial pathway and the death receptor pathway, by directly inhibiting terminal effector caspase-3, caspase-7, and caspase-9 activity[1,2]. Thus, it inhibits apoptosis pathway differently from bcl-2, which blocks mitochondrial cytochrome c release into the cytosol, resulting in the inhibition of mitochondrial apoptotic pathway. Survivin is expressed during embryonic and fetal development but is undetectable in terminally differentiated normal adult tissue. However, it is re-expressed in transformed cell lines and several human cancer cells at a frequency of 34–100 % [3,4]. As a prognostic factor, survivin expression is significantly associated with poor clinical outcome in cancers, such as neuroblastoma, colorectal cancer, breast cancer, lung cancer and esophageal cancer [5-10].

Survivin expression in pancreatic cancer has not been widely studied. Satoh et al reported that survivin was expressed in malignant portion of intraductal papillary-mucinous tumor (IPMT) but not in chronic pancreatitis, and thus proposed that survivin expression may be upregulated at an early stage of tumorigenesis by reducing cancer cell apoptosis [11]. In another study, Asanuma et al reported that survivin acts as an inducible radioresistance factor in pancreatic cancer cell line [12].

In the present study, we investigated survivin expression in pancreatic cancer and its association with clinical outcome.

## Methods

### Patients and specimens

Formalin-fixed, paraffin-embedded blocks from forty-nine surgically resected pancreatic tumor tissues were studied. All samples were collected under protocols approved by the local Institutional Review Board (IRB). All patients were diagnosed with pancreatic cancer at Kangnam St. Mary's hospital between May 1994 and November 2001. Patients' clinicopathological characteristics are summarized in Table 1 &2. Median age was 59 years (range, 33–76 years). According to AJCC cancer stage system (4^th ^ed, 2002), 34 patients (69.4%) had stage I and II disease, and 10 patients (20.4%) had stage III. disease. Fourteen patients had only palliative resection and treated combination chemotherapy with epirubicin, cisplatin and 5-FU. Of 35 patients who had curative surgery, 20 patients (40.8%) underwent adjuvant treatment with radiotherapy. Recurrent cancer developed in 18 patients, and the median time to recurrence was 156 days (range, 58–1499 days).

**Table 1:** Patients' characteristics

		No. of case
Age*		59 (33–76)

M:F		36:13

Chief Complaint	Pain	20(40.8%)
	Jaundice	18(36.7%)
	Indigestion	6(12.2%)
	Others	5(10.1%)

Location	Head	35(71.4%)
	Body	1(2.0%)
	Tail	7(14.3%)
	Body & Tail	6(12.3%)

Stage	I	8(16.3%)
	II	26(53.1%)
	III	10(20.4%)
	IV	5(10.2%)

Adjuvant treatment	Yes	20(40.8%)
	No	14(28.6%)

Recurrence after curative surgery		18/35^+^

*: median,

+: number of recurrence/total number of operable cases

**Table 2:** Pathological characteristics

		number
differentiation	well	8 (16.3%)
	moderate	34 (69.4%)
	poorly	3 (6.1%)
	others	4(8.2%)

lymphatic invasion	+	28 (57.1%)
	-	18 (36.7%)

vein invasion	+	10 (20.4%)
	-	36 (73.5%)

perineural invasion	+	34 (69.4%)
	-	12 (24.5%)

### Tissue microarray

To construct the tissue microarray block, small core biopsies were taken from non-necrotic, morphologically representative areas of paraffin-embedded tumor tissues and assembled on a recipient paraffin block. This was performed using a precision instrument (Micro Digital Co. Korea). The biopsied core was 3.0 mm in diameter, which was sufficient for assessing the morphological features in the tissues, and then 30 cores were assembled on a recipient paraffin block. After construction, 5 μm sections were cut and hematoxylin-eosin staining was performed on the initial slide to verify the histology.

### Immunohistochemistry

Immunohistochemical staining was performed on 5 μm sections of the tissue microarray blocks. Paraffin sections were mounted on superfrost glass slides, deparaffinized and rehydrated in a graded ethanol series, and then subjected to microwave antigen retrieval. Endogenous peroxidase activity was blocked using 0.3% hydrogen peroxide. Sections were incubated for l hour at room temperature or at 4°C overnight with the following primary antibodies at the dilutions specified: survivin Ab-6(NeoMarkers, CA, USA) diluted 1:50, p53 (DAKO Corporation, Carpinteria, CA, USA) diluted 1:100, Bax (DAKO) diluted 1:200. Immunohistochemical staining was performed using the rabbit or mouse DAKO ChemMateTM EnVisionTM system, and the Peroxidase/DAB Kit (DAKO). Sections were then counterstained with Mayer hematoxylin and then dehydrated, cleared and mounted.

Results were interpreted by two independent pathologists who were blinded to the specific diagnosis and prognosis for each case. Tumor cells that showed distinct nuclear or cytoplasmic staining were considered positive. Immunohistochemical staining was scored on a three-tiered scale: score 0 = less than 10% of cells positive; 1 = 10 – 49% positive; score 2 = ≥50% positive.

### Statistical analysis

The SPSS (version 11.0) statistical package was used to analyze the data. Using the Chi-square Test and the Fisher's exact test, the immunohistochemical profiles were compared with clinico-pathologic parameters.

## Results

### Expression of survivin, p53, and Bax

**Figure 1:**
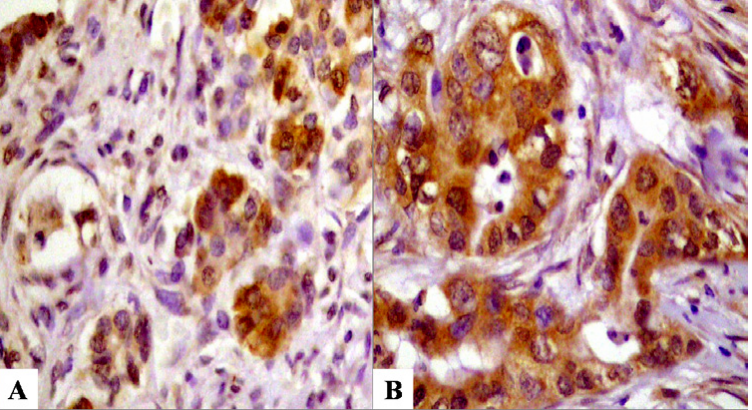
**Immunohistochemical staining for surviving. **Strong cytoplasmic expression for survivin in pancreatic cancer gland (A: × 100, B: × 400).

**Figure 2:**
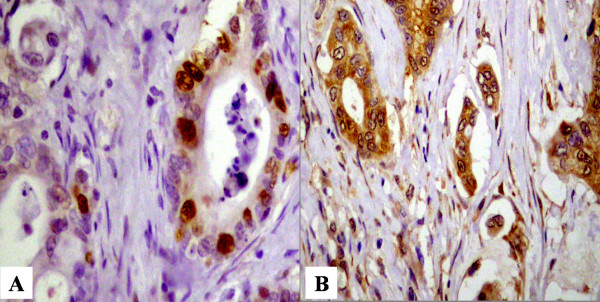
**Immunohistochemical staining for p53 and Bax. **A: Strong positive expression of p53 in cancer cell (× 400). B: Positive expression of Bax in pancreatic cancer cell(× 200).

Of forty-nine cases of pancreatic cancer, 46 cases (93.9%) were positive for survivin expression (Fig. 1). In immunohistochemical staining for p53 and Bax, sixteen cases (32.7%) and thirty-eight cases (77.5%) were positive, respectively (Fig. 2A & B). These are summarized in Table 3. Survivin expression was not associated with p53 or Bax expression (p = 0.9969 and 0.0931, respectively, Table 4)

**Table 3:** The result of immunohistochemical staining

	Survivin	p53	Bax
Score 0	3(6.1%)	33(67.3%)	11(22.4%)
Score 1	17(34.7%)	9(18.4%)	11(22.4%)
Score 2	29(59.2%)	7(14.3%)	27(55.1%)

**Table 4:** Survivin expression and p53, Bax in pancreatic cancer

		p53 (p = 0.9969)			Bax (p = 0.0931)		
			
		0	1	2	0	1	2
survivin	0	2	0	1	2	1	0
	1	12	3	2	6	2	9
	2	19	6	4	3	8	18

### Survivin, p53, and bax expression and clinico-pathologic characteristics

Of 49 tissue specimens, only tumor tissues were obtained from 3 patients due to far advanced stage. We evaluated pathological characteristics in 46 patients. There was no correlation between disease stage and expression of survivin, p53, or bax protein (Table 5), and no correlation was found between survivin expression and p53 or bax protein expression. However, perineural invasion was more common in the survivin-positive, and venous invasion was more common in the survivin- negative group (p = 0.041 and 0.040, respectively, Table 6).

**Table 5:** Stage and survivin, p53, bax expression

		Stage I	Stage II	Stage III	Stage IV	p value
Survivin	0	0	2	0	1	0.386
	1	3	7	5	2	
	2	5	17	5	2	

p53	0	5	18	7	3	0.207
	1	1	4	3	1	
	2	2	4	0	1	

Bax	0	4	5	2	0	0.414
	1	2	6	1	2	
	2	2	15	7	3	

**Table 6:** Pathological parameters and survivin expression

	Lymphatic invasion (p = 0.094)		Venous invasion (p = 0.040)		Perineural invasion (p = 0.041)	
	
	Yes	No	Yes	No	Yes	No
0	1	1	1	1	2	0
1	12	5	3	14	12	5
2	15	12	6	21	20	7

### Clinical outcome and survivin expression

**Figure 3:**
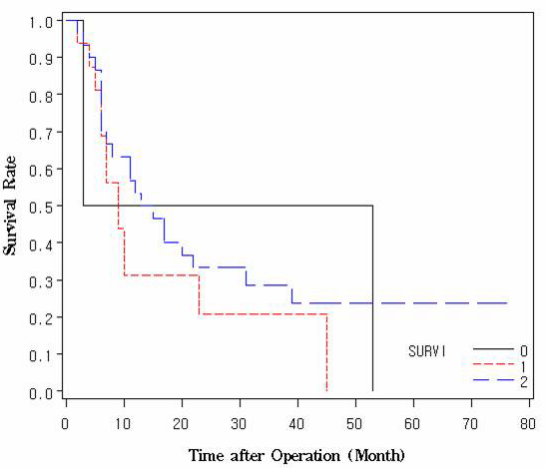
**Overall survival. **No significant difference in overall survival according to intensity of survivin expression.

We evaluated the association between survivin expression and survival. We did not find any significant difference correlation between survival and survivin expression (Fig 3) because only three patients showed survivin negative. It was difficult to identify the association due to the vast majority of patients expressing survivin.

Fourteen patients received epirubicin, cisplatin and 5-FU combination chemotherapy as initial treatment for advanced stage of disease. Responsiveness to chemotherapy appeared to be slightly better in patients with low survivin expression compared to those with high expression (28.8% vs.7.1%, respectively; Table 7), although there was no statistically significant correlation. We also investigated the efficacy of chemotherapy and Bax expression, but all patients who treated with chemotherapy showed 2+ in Bax expression. Therefore, we did not analyze statistical significance.

**Table 7:** Responsiveness to chemotherapy and survivin expression

		Responder	Non-responder
survivin	Score 1	4 (28.8%)	4 (28.6%)
(p = 0.2098)	Score 2	1 (7.1%)	5 (36. 7%)

## Discussion

Survivin, an IAP, has been studied as a prognostic marker in various cancers. Adida et al reported that survivin expression in neuroblastoma correlated with unfavorable histology, aggressive and disseminated disease [5]. In colorectal cancer and breast cancer, patients with survivin-positive tumors had a decreased apoptotic index and worse survival rates than those with survivin-negative tumors [7,8]. In addition, survivin expression correlated with poor prognosis in esophageal cancer and non-small cell lung cancer [9,10].

The clinical significance of survivin expression in pancreatic cancer is not well understood. Previous studies reported that survivin was expressed at an incidence of 77%–88% [11,13]. In our study, survivin was expressed in 93.9%, and was highly expressed in most cases (59.2%). In pathological parameters, neural invasion was more common in survivin positive group. Neural invasion is a specific metastatic route in pancreatic cancer and perineural invasion is known to be a prognostic marker for the recurrence of pancreatic cancer after surgery [14]. However, venous invasion was more common in survivin negative group. Venous invasion in pancreatic cancer can be a risk factor for liver metastasis [15]. We did not explain this discrepancy, but we suggested that venous invasion may be an independent prognostic factor irrelevant to survivin expression. Recent study reported that survivin expression has different clinical significance according to expression pattern [16]. Although we did not distinguish the expression pattern, it may be another explanation for this discrepancy. It is important to predict recurrence or metastatic pattern after curative surgery because of determining adequate adjuvant treatment modality in pancreatic cancer, so further study may be warranted to investigate the association between survivin and pathological parameters.

We did not find any other association between survivin expression and clinical course due to the limitation of patients enrolled in this study. Most patients in our study were at an early stage, making it difficult to compare survivin expression according to various stage. Secondly, the vast majority of pancreatic cancer tissue in the present study expressed survivin, meaning survivin-negative group was very small. For the same reason, we could not determine that survivin expression was a prognosis factor. Recently, Kami et al reported that survivin expression may be a prognostic factor in pancreatic cancer. In that study, 63.8% of patients showed survivin expression, resulting in a good proportion of survivin-negative patients for comparison [17]. We also investigated whether survivin expression was associated with poor clinical outcome, including recurrence and survival, but no significant correlation was observed. However, It is not determined that survivin may not be associated with poor clinical outcome because of limitation of our study. It may be that further study involving great number of patients is required in order to determine the clinical significance of survivin expression in pancreatic cancer.

It is not certain whether survivin expression is a predictive marker for anticancer therapy such as radiotherapy or chemotherapy in pancreatic cancer. In a study using a pancreatic cancer cell line, Asanuma et al reported that survivin may be a radioresistant factor [12], and Kato et al suggested that survivin expression may be a predictive marker for chemotherapy as well as prognostic marker in esophageal cancer in their previous study [9]. We examined the survivin expression in fourteen patients treated with chemotherapy to evaluate the potential of survivin as a predictive marker for chemotherapy. Although the findings were statistically not significant, there was a suggestion that non-responder group showed high survivin expression. On the basis of this finding, we hypothesize that survivin is a potential marker for predicting responsiveness to chemotherapy.

The p53 mutation is frequently seen in pancreatic cancer, and its expression correlates with overall survival after pancreatectomy [18]. Previous study found survivin and p53 may play important roles in the transition from adenoma to carcinoma in situ in IPMT, and that survivin expression was higher in p53-positive pancreatic cancers [18,19]. In the present study, while 34.6% of tumors expressed p53, there was no correlation between p53 and survivin expression. Bcl-2 family proteins also play important roles in regulation of apoptosis. Previous work showed Bax expression was associated with a better prognosis and was found to be a predictive marker for adjuvant chemotherapy [20]. In the present study, while 84.6% of tumors expressed Bax, there was no correlation between its expression and survivin expression or clinico-pathological parameters. The present data did not indicate that p53 or Bax protein correlated with survivin expression in pancreatic cancer. It is not certain why this discrepancy exists, although all these proteins may block apoptotic pathway. Whether p53 or Bax expression in pancreatic cancer is related to prognosis is still under debated. In addition, some authors suggest that survivin may be independent prognostic marker not associated with p53 or Bax expression [21,22]. Therefore, we hypothesized that tumor cell may have other pathway to evade apoptosis after Bax or p53-involved steps in pancreatic cancer. It appears further large scale studies are required to investigate any correlation between survivin and cell cycle regulatory protein expression in pancreatic cancer.

## Conclusion

In summary, survivin was expressed at high levels in the vast majority of pancreatic cancer. While the data did not indicate any correlation between survivin expression and clinical outcome, they may be interpreted as suggesting survivin may be a predictive marker in pancreatic cancer for anticancer therapy in pancreatic cancer. We believe further prospective studies are warranted to determine whether survivin is a prognostic or predictive marker in pancreatic cancer.

## Competing interests

The author(s) declare that they have no competing interests.

## Authors' contributions

Lee MA conceived of the study and carried out design and analysis. Park GS, Lee JH, and Jung JH carried out immunohistochemical staining and reviewed the all pathological data. Kang JH, Hong YS and Lee KS participated in coordination and helped to draft the manuscript. Kim SN and Kim DG supplied tissue specimen and collect clinical data.

## Pre-publication history

The pre-publication history for this paper can be accessed here:


